# The Comparison of Caring Attributes Among Undergraduate and Postgraduate Nursing Students: A Cross‐Sectional Study

**DOI:** 10.1002/hsr2.71001

**Published:** 2025-07-09

**Authors:** Behnaz Bagherian, Monirsadat Nematollahi, Roghayeh Mehdipour‐Rabori, Shima Mehrabian, Peiman Parandeh Afshar, Asma Ghonchehpour

**Affiliations:** ^1^ Razi Faculty of Nursing & Midwifery Kerman University of Medical Sciences Kerman Iran; ^2^ Reproductive and Family Health Research Center Kerman University of Medical Sciences Kerman Iran; ^3^ Nursing Research Center Kerman University of Medical Sciences Kerman Iran; ^4^ Student Research Committee Kerman University of Medical Sciences Kerman Iran

**Keywords:** caring attributes, education, nursing students

## Abstract

**Background and Aims:**

Caring attributes reflect the nurses' view of care and the importance of caring roles for nurses. Nursing education also provides an ideal situation to develop students' caring behaviors. However, nursing educators have little knowledge of the influences of educational programs and student development at different stages, mainly due to limited exposure to updated training or research in this area. The present study aimed to compare the caring attributes experienced by nursing students at undergraduate and postgraduate levels.

**Methods:**

This a descriptive cross‐sectional study was conducted among 200 nursing students in the Faculty of Nursing and Midwifery of Kerman University of Medical Sciences in Kerman, Southeastern Iran, from 2019 to 2020. Participants were selected using stratified random sampling. The data were collected using a two‐part questionnaire including a demographic information form and a 34‐item caring attributes questionnaire (CAQ).

**Results:**

It was shown that the mean score of students' caring attributes was 122.03 ± 11.77 at a desirable level (higher than the median score (= 104.5). No statistically significant difference in overall caring attribute scores was found among students across the three educational levels (Bachelor's, Master's, and Doctoral) (*p* = 0.27). But at the undergraduate level with eight semesters, nursing students in the fifth semesters have the highest caring attributes (*p* = 0.04). Also, the undergraduate students had higher scores on the caring negation compared to the master students and PhD students (*p* = 0.03). The PhD. students scored significantly higher on the scores of caring communication compared to the undergraduate and master students (*p* = 0.009).

**Conclusions:**

While overall caring attributes were at a desirable level, the study revealed significant differences in specific attributes based on educational level. These findings highlight the importance of considering educational level when fostering caring behaviors in nursing students, particularly in communication and negation aspects of care.

## 1 Introduction and Background

Caring as the basis and essence of the nursing profession [1] refers to having the knowledge to examine the patient's needs, provide scientific solutions to meet these needs, and provide comfort to the patient [2]. Some researchers consider caring to consist of the two physical (instrumental) and expressive aspects [1]. Instrumental care provides physical health and comfort and expressive care meets the patient's psychosocial needs, induces a sense of hope and value in the patient, and facilitates the patient's recovery [3]. Physical health and mental health are inseparable and physical (instrumental) and expressive caring behaviors play the same role and have the same level of significance in providing personal health [4].

Care is an important indicator for evaluating the quality of nursing. Caring experiences increase the nurse's personal and professional satisfaction and increase the quality of nursing care. Patients who have had caring encounters with nurses have reported greater satisfaction, more effective coping skills, increased adaptability, and decreased anxiety [5, 6, 7]. Currently, the dissatisfaction of service recipients with the service provided by health centers has become a global problem [2], which indicates the negligence of health care staff and their non‐caring attitudes and [1, 8, 9] nurses must have the knowledge and skills to provide context‐based care to meet each patient's unique needs. Nurses are expected to acquire this ability while undergoing basic nursing education [10]. Besides, given that caring is associated with positive outcomes for the patient, the nurse, and the organization, nursing students' eligibility for providing optimal care must be guaranteed upon graduation from nursing education programs and training students who are qualitatively suitable for this job [1]. Achieving this goal requires a review and focus on the nursing education program to ensure a balance between the formation of work value bases and the acquisition of clinical skills by learners in this field [11].

The majority of nurses enter the profession because of their humanitarian beliefs and aim to provide standard care. However, it is not clear how and to what extent the core and professional values are formed during the nursing education program [12]. Phillips et al. (2015) showed that new nursing students are eager to learn care and make positive changes in the patient and his/her family [11]. However, Loke et al. (2015) who studied caring behaviors of nursing students showed that these behaviors undergo significant decreases in nursing students from the first year to the last year of nursing education and this decrease is greater among students in the final semester [3]. Bagnall et al. (2018) showed that Inner care is a personal quality necessary for holistic care. Therefore, it is important that nursing graduates have an adequate level of internal care to demonstrate care competence. Nursing educators must ensure that future nursing graduates possess a high level of nursing competence to encourage best nursing practice [13]. Li et al. (2016) concluded that there were no significant differences in caring behaviors between nursing students and registered nurses. Therefore, role models, combining different teaching strategies by nursing faculty should be provided to nursing students to develop appropriate caring behaviors [14]. However, Guo et al. (2013) have recently stated that there is an international need to push all educational programs toward an approach based on the development of human values, and they successfully applied several creative educational models to facilitate the development of caring qualities among nursing students in Asia [15].

The development of nursing students and their successful transfer to a caregiver profession depends on the relationship between the preceptor, the student, and the educational environment. In fact, the interaction that develops between students and preceptors helps students grow as caregivers. Therefore, nursing education provides an ideal situation to develop, increase, and promote students' caring behaviors [16]. Nevertheless, nursing educators have very little knowledge of the impact of educational programs at different stages on nursing students and how they progress and develop during these programs [17]. Accordingly, the present study aimed to compare the caring attributes experienced by nursing students at undergraduate and postgraduate levels.

## 2 Methods

### 2.1 Study Design and Setting

The present study was a descriptive cross‐sectional study. This study was conducted in the Kerman University of Medical Sciences in Kerman, Iran. Kerman is the largest city in the southeast of Iran. This study was conducted in one of the faculties of Kerman University of Medical Sciences named Razi school of Nursing and Midwifery. This faculty is the oldest school of Nursing and Midwifery in southwest Iran. The participants were undergraduate and postgraduate nursing students such as second year nursing master's students and PhD nursing students of Kerman University of Medical Sciences in the academic year 2019–2020. In the educational year of 2019–2020, 399 nursing students were studying in this faculty.

### 2.2 Sampling Procedure

The sample size was estimated as 193 students based on the total number of nursing students in the School of Nursing and Midwifery of Kerman University of Medical Sciences using the following formula:

$$n=(z^{\left(2\text{pq}\right)}/d^{2})/(1+1⁄N\;((Z^{2}\text{pq})⁄d^{2}-1)).$$

However, given the probability of dropout, 200 students were selected as the participants in the research sample. The participants were selected using stratified sampling. To this end, each academic level was considered as a stratum and the number of students selected from each stratum was determined in proportion to the strata size. The total number of students at the time of the present study and the estimated number of samples from each stratum were as follows:

The total number of undergraduate nursing students was 235/the number of undergraduate students in the sample of the present study was 114, The total number of nursing students in the master's degree was 146/the number of master students in the sample of the present study was 75, The total number of PhD students in nursing was 18/Number of PhD students in the sample of the present study was 11.

Afterward, the students meeting the inclusion criteria were selected using convenient sampling in each stratum. Inclusion criteria included enrollment in one of the undergraduate, graduate and doctoral programs in nursing at Kerman University of Medical Sciences and willingness to participate in the study. Incomplete questionnaires were excluded from the study. A total of 150 questionnaires were distributed among the undergraduate nursing students, 75 questionnaires among the master's degree students and 11 questionnaires among of PhD students in nursing at the School of Nursing and Midwifery of Kerman University of Medical Sciences to complete, and 146 questionnaires from undergraduate nursing students, 49 questionnaires from master's degree students and 5 questionnaires from PhD students were returned (a response rate of 84.74%).

### 2.3 Participant Selection

Upon the approval of the research project, the researcher referred to the university and presented a written letter of introduction to the management and security department of the university. The researchers visited the library, study hall, student dormitories, and classrooms in the School of Nursing and Midwifery of Kerman University of Medical Sciences in the mornings and evenings for 4 weeks and distributed the questionnaires to students upon obtaining informed consent. The students completed the questionnaires in a self‐report manner and returned them to the researchers. The participants were selected from among students studying in undergraduate and postgraduate nursing programs at Kerman University of Medical Sciences who were willing to participate in the study. The students were assured that participation or nonparticipation in this study will not affect their educational process and sampling was not done by the professors so that the student participates based on her interest and desire and is not influenced by the teachers' determinism.

### 2.4 Instruments

The study assessed specific caring attributes, including care communication, caring as advocacy, care negation, care essence, and care education, using the 34‐item Caring Attributes Questionnaire (CAQ). Each of these subscales evaluates a distinct aspect of caring behavior in nursing students.

The instrument to collect the data was a two‐part questionnaire including a demographic information form and a 34‐item CAQ.

### 2.5 Demographic Information Questionnaire

The first section of the questionnaire assessed the participants' personal characteristics including age, sex, education, admission year, marital status, and occupation.

### 2.6 Caring Attributes Questionnaire

To assess the caring attributes of the nursing students, the CAQ was used. This 34‐item questionnaire was developed to measure professional self‐concept, caring attributes, and technological influences for use in an international sample of nurses (Arthur et al., 1999). The main version of this tool consists of three subscales (60 items assessing caring attributes, 14 items measuring technological influences on nursing care, and 30 items assessing technological influences in different parts of the hospital). Each item is scored using a five‐point Likert scale (1 = disagree, 2 = somehow disagree, 3 = undecided, 4 = somehow agree, and 5 = agree). The 60‐item CAQ was developed based on a review of the literature, and Arthur et al. analyzed its psychometric properties in an international study and reduced the items to 31 which has four subscales: care communication (Cronbach's alpha = 0.84); caring as advocacy (Cronbach's alpha = 0.78); engagement in care (Cronbach's alpha = 0.79) and care education (Cronbach's alpha = 0.62) [18, 19].

### 2.7 Validity and Reliability of Questionnaires the Persian Version of Caring Attributes Questionnaire

The Persian version of the 31‐item CAQ was prepared in this study using forward and backward translation. First, two health care experts translated the English version of the questionnaire into Persian independently. Afterward, another translator who had never seen the original questionnaire translated the Persian version into English. The original and translated versions of the questionnaire were analyzed semantically, grammatically, empirically, and conceptually by a panel of bilingual experts and any discrepancies were resolved. This step was aimed at determining whether the translated version had the same content as the original. To check the content validity of the questionnaire, it was reviewed by ten professors of the School of Nursing and they examined the items in terms of simplicity, clarity, and relevance. One item which was culturally different (Touching the patient when comfort is needed) was removed by the experts and four items were added to this questionnaire. Finally, the 34‐item CAQ was developed and administered to the participants in this study. Factor analysis of these 34 items identified five subscales including care communication (include Item numbers 1, 6, 8, 9, 10, 11); caring as advocacy (include Item numbers 13, 14, 15, 17, 18, 20, 21, 22, 23, 25, 27, 28); care negation (include Item numbers 4, 5, 7, 12, 19, 26, 29, 31), care essence (include Item numbers 2, 3, 16, 30), and care education (include Item numbers 32, 33, 34). The total score on the questionnaire is calculated as the mean of the scores for all 34 items. Items 4, 5, 7, 12, 16, 19, 31, and 34 are scored inversely. The range of scores in this questionnaire varies from 34 to 170, with a higher score indicating better caring attributes and a more positive attitude towards care.

Caring communication involves active listening, conveying empathy, and clear information exchange, which are essential for building therapeutic relationships. Caring as advocacy involves supporting and speaking up for patients' needs, rights, and preferences, ultimately promoting patient‐centered care. Caring negation describes the behaviors that deny or diminish the patient's perceived needs or wishes, often resulting in a lack of empathy, support, or advocacy in the nurse‐patient relationship. Caring essence refers to the fundamental and intrinsic nature of caring in nursing, embodying core values such as compassion, empathy, and genuine concern for the patient's well‐being. It represents the authentic, heartfelt aspect of care that underpins all caring actions and interactions. Care education encompasses the teaching of essential skills and ethical principles that underpin compassionate and effective nursing practice.

To assess the reliability of the questionnaire, it was administered to 20 nursing students and the internal consistency of the items was calculated using Cronbach's alpha coefficient. The corresponding value was 0.84, indicating the good reliability of the tool for assessing the caring attributes of nursing students in Iran.

### 2.8 Data Analysis

The collected data were analyzed using SPSS software (version 25). Descriptive statistics (frequency, percentage, mean, and standard deviation) were used to describe the participants' demographic characteristics. Also, the data collected from each subscale were analyzed to assess the levels of caring attributes across different domains, using descriptive statistical methods. Furthermore, inferential statistics including the Mann–Whitney U test, Kruskal Wallis test, and Spearman correlation test were used to assess the relationship between the participants' demographic characteristics and their caring attributes. Univariate linear regression was used to examine the variables predicting the total score of Caring attributes in the students.

### 2.9 Ethics Approval and Consent to Participate

This study was confirmed under the code of ethics IR.KMU.REC.1395.721 and was approved by the Ethics Committee of Kerman University of Medical Sciences. All methods were carried out in accordance with relevant guidelines and regulations. The students were told that their participation in the study was voluntary and they could leave it if they wished. Besides, they were ensured the data collected would be kept confidential and would only be used for the research objectives and providing better solutions. Besides, informed written consent was obtained from the participants.

## 3 Results

### 3.1 Socio‐Demographic Characteristics

In total, 200 students were assessed. The results of data analysis indicated that the participants' mean age and their clinical work experience were 24.28 ± 6.50 and 2.08 ± 3.43, respectively. Besides, 32.5% of the participants were studying in the second semester and 73.0% of them were studying at the undergraduate level. The majority of participants (67.5%) were female and single (66.5%) and 41.0% of them had clinical work experience. The results showed that there was a significant relationship between gender in all students and female students had have the highest caring attributes. Also, Tukey's HSD test showed that at the undergraduate level with eight semesters, nursing students in the fifth semesters have the highest caring attributes (Table 1).

**Table 1:** The distribution of the participants in terms of the demographic characteristics and their association with caring attributes in 200 students.

Variable	Category	*N* (%)	Caring attributes		Statistical test	*p* value
			Mean	SD		
Gender	Female	135 (67.5)	123.65	10.76	Z = −2.67	0.008
	Male	65 (32.5)	118.68	13.09		
Marital status	Single	133 (66.5)	120.88	13.06	Z = −1.35	0.17
	Married	67 (33.5)	124.31	8.28		
Clinical experience	Yes	82 (41)	123.18	12.65	Z = −1.17	0.24
	No	118 (59)	121.24	11.11		
Education	Undergraduate	146 (73)	121.23	12.60	H = 2.61	0.27
	Master	49 (24.5)	123.84	8.85		
	PhD	5 (5)	128.00	9.54		
Undergraduate semester	2	35 (24.0)	120.80	14.81	H = 12.86	0.04
	3	3 (2.1)	104.00	0.01		
	4	42 (28.8)	120.93	10.12		
	5	—	—	—		
	6	32 (21.9)	122.28	7.40		
	7	—	—	—		
	8	34 (23.3)	122.59	16.21		
Master semester	2	30 (61.2)	121.97	7.85	H = 9.44	0.05
	4	12 (24.5)	124.83	10.29		
	6	7 (14.3)	130.14	8.29		
PhD semester	5	2 (40)	131.00	2.82	Z = 0.09	0.77
	7	3 (60)	126.00	12.70		
Teaching experience	Yes	16 (8.0)	123.87	8.40	Z = −0.35	0.72
	No	184 (92)	121.88	12.03		
		Mean ± SD	Spearman's rho *p* value			
Age		24.28 ± 6.50			r = 0.13	0.06
Clinical work experience		2.08 ± 3.43			r = 0.07	0.35

Abbreviations: H, Kruskal Wallis Test; r, Spearman's Rank Correlation Coefficient; Z, Mann‐Whitney U Test.

### 3.2 The Comparision of Caring Attributes and Its Dimensions in Terms of the Students' Educational Level

It was shown that the overall mean score of students' caring attributes was 122.03 ± 11.77. This was higher than the median score (= 104.5) and at a desirable level (The range of scores in this questionnaire varies from 34 to 170). The mean score for the Caring essence domain was 15.40 ± 2.22 (Max = 20.00, Min = 4.00), the Caring communication domain was 25.76 ± 3.93 (Max = 30.00, Min = 6.00), the Caring advocacy domain was 48.93 ± 6.98 (Max = 60.00, Min = 12.00), the Caring education domain was 10.47 ± 2.30 (Max = 15.00, Min = 5.00) and the Caring negation domain was 17.60 ± 5.30 (Max = 36.00, Min = 9.00). The mean scores for all five dimensions of caring attributes were higher than the median scores.

The results showed that there were no significant difference between caring attributes score and students' educational level (*p* = 0.27). But the PhD students scored significantly higher on the scores of caring communication compared to the undergraduate and master students. Also, the undergraduate students had higher scores on the caring negation compared to the master students and PhD students (*p* = 0.03) (Table 2).

**Table 2:** The comparision of caring attributes and its dimensions in terms of the students' educational level.

Variable	Statistics	Caring advocacy	Caring essence	Caring education	Caring negation	Caring communication	Caring attributes
Undergraduate	Mean rank	95.25	102.16	96.79	105.64	94.00	98.04
Master	Mean rank	112.84	92.06	106.39	90.74	114.21	103.93
PhD	Mean rank	133.00	134.70	151.10	46.10	155.90	138.60
	Statistical test	H = 5.03	H = 3.00	H = 5.04	H = 7.00	H = 9.34	H = 2.61
	*p* value	0.08	0.22	0.08	0.03	0.009	0.27

Abbreviation: H, Kruskal Wallis Test.

### 3.3 Caring Attributes and It Association With Demographic Characteristics in Terms of the Students' Educational Level

A comparison of the mean total score of the students' caring attributes indicates that the female undergrduate students had significantly higher scores compared to the male students (Table 3). Also, multivariate linear regression was used to examine the variables predicting the total score of Caring attributes in students, and the regression results showed that there was no statistically significant relationship with demographic variables (*p* > 0.05).

**Table 3:** Caring attributes and it association with demographic characteristics in terms of the students' Educational level.

Variable	Category	Undergraduate			Master			PhD		
		*N* (%)	Caring attributes	Statistical test (*p* value)	*N* (%)	Caring attributes	Statistical test (*p* value)	*N* (%)	Caring attributes	Statistical test (*p* value)
			Mean rank			Mean rank			Mean rank	
Gender	Female	88 (60.3)	81.15	Z = −2.69 (0.007)	43 (87.8)	25.01	Z = −0.01 (0.9)	4 (80)	3.13	Z = −0.36 (0.7)
	Male	58 (39.7)	61.90		6 (12.2)	24.92		1 (20)	2.50	
Marital status	Single	121 (82.9)	70.97	Z = −1.59 (0.11)	12 (24.5)	27.29	Z = −0.64 (0.52)	0	00	—
	Married	25 (17.1)	85.76		37 (75.5)	24.26		5 (100)	3.00	
Clinical experience	Yes	34 (23.3)	74.24	Z = −0.12 (0.9)	43 (87.8)	26.29	Z = −1.70 (0.09)	5 (100)	3.00	—
	No	112 (76.7)	73.28		6 (12.2)	15.75		0	0.00	
Teaching experience	Yes	2 (1.4)	67.25	Z = −0.21 (0.84)	9 (18.4)	18.83	Z = −1.44 (0.15)	5 (100)	3.00	—
	No	144 (98.6)	73.59		40 (81.6)	26.39		0	0.00	
		**Mean (SD)**	**Spearman s rho**	***p* value**	**Mean (SD)**	**Spearman s rho**	***p* value**	**Mean (SD)**	**Spearman s rho**	***p* value**
Age		21.27 (2.84)	0.12	0.14	31.35 (5.66)	0.09	0.55	43.20 (6.07)	−0.46	0.43
Clinical work experience		1.11 (2.57)	0.03	0.73	4.49 (4.08)	0.08	0.60	6.60 (3.78)	−0.37	0.54

Abbreviations: Spearman s rho, Spearman's Rank Correlation Coefficient; Z, Mann‐Whitney U.

## 4 Discussion

Having caring attributes is a requirement of quality nursing care. Caring attributes should be promoted in nursing students during holistic care programs to provide quality care as improving the quality is associated with positive outcomes for patients, nurses, and the organization as a whole [20].

The present study indicated that the scores of caring attributes and their dimensions were higher than median for students at the undergraduate and postgraduate programs. However, the results of Machul et al. (2022) study showed that the caring ability inventory score considered low in the Polish nursing students [21]. Also, in another study, Machul et. al. (2023) found that the caring ability inventory score was low in the Polish nurses [22]. One of the reasons for this discrepancy is that the tool used in the two studies were different (CAI = caring ability inventory). No statistically significant difference in overall caring attribute scores was found among students across the three educational levels (Bachelor's, Master's, and Doctoral). Hosseinzadeh et al. (2019) found that there is no significant difference between nurses with bachelor's, master's, and doctoral degrees in terms of caring behaviors [23]. In a similar way, a study conducted by Zamanzadeh et al. (2014) in Tabriz and Urmia using the Q‐CARE questionnaire showed perceptions of caring behaviors were not significantly different between the students at lower and higher academic levels. Besides, most of the students mentioned technical and professional caring behaviors as more important than the other caring behaviors [24]. However, Atashzadeh et al. (2015) suggested that the caring attributes of students at a higher academic level were lower than students at lower academic levels [25]. One of the reasons for this discrepancy is that the present study focused on undergraduate and postgraduate students. Besides, the tools used in the two studies were different.

Besides, the PhD students scored significantly higher on caring communication compared to the undergraduate and master students. Accordingly, it can be suggested that studying at higher academic levels improves nurses' communication toward care. The higher caring communication scores among PhD students likely reflect their advanced training in interpersonal skills and research. Similarly, the undergraduate students gained higher scores on the caring negation than other students. The elevated caring negation scores in undergraduates might stem from their initial exposure to clinical realities and developing understanding of the complexities of care.

The present study also showed that undergraduate students in the fifth semesters (3rd‐year‐between second to eighth semesters) have the highest caring attributes. However, Machul et al. (2022) study showed thar year of study significantly correlated with overall caring ability, with MA 1st‐year students scoring highest and BA 3rd‐year students lowest [21]. Loke et al. (2015) suggested that caring behaviors decreased significantly from the first to the last semester and this decrease was higher in the last semester students [3]. Explaining this decline, Phillips et al. (2015) stated that newly admitted students are eager to learn caring and change patient conditions and that the educational process decreases students' caring behaviors [11]. In the interpretation of the obtained results, it can be mentioned that, considering that each year of nursing bachelor's degree includes two semesters. Before conducting the study, it was thought that the nursing students of the final year (seventh and eighth) had a higher score of care changes compared to the lower semesters, but this score in the final semester (the eighth semester), was far lower than other academic semesters. Meanwhile, students of the seventh and eighth semesters are trained independently and interned in the hospital, and it is expected that caring attributes will increase with the increase of their experience in the hospital and the acquisition of practical skills. Perhaps the reason can be found in the lack of theory lessons in the final year, the independence of students and the acquisition of education from the hospitals, and the conflicts between theory and practical nursing. In the higher semesters, students are mostly in the hospitals and the gap between theory and nursing practice can lead to conflicts in the student who takes refuge in the clinical experiences of his colleagues to solve these conflicts and uses less of his up‐to‐date knowledge.

A comparison of the mean total score of the students' caring attributes in the present study showed that the female students had significantly higher scores on all dimensions compared to the male students, and the largest difference was related to caring advocacy. Similarly, Hosseinzadeh et al. (2019) showed that women had a higher score for caring behaviors than men [23]. In contrast, Atashzadeh et al. (2015) who studied nursing students' views on the importance of caring behaviors at the beginning, middle, and end of the undergraduate program stated that the male and female students' views were not different [25]. One of the reasons for this discrepancy is that the present study focused on undergraduate and postgraduate students. Besides, the tools used in the two studies were different.

The present study also showed that there was not a statistically significant difference between the single and married students in terms of the total scores of caring attributes, and the married students gained higher scores for these dimensions. Hosseinzadeh et al. (2019) showed that the two married and single groups did not differ significantly in terms of caring behaviors [23].

The findings of the present study showed that, there was no significant difference between caring attributes and clinical working experience. The results of Machul et al. (2022) study showed the same results in nursing students [21]. However, Phillips et al. (2015) reported that people who had a history of caring work or completed nursing courses before university admission had better caring attributes than others [11]. Moreover, the educational process is different in various communities and health education centers, which can be another reason for this discrepancy.

The present study showed no significant relationship between age and caring attributes. But, Atashzadeh et al. (2015) showed a negative correlation between age and caring attributes, and younger people reported higher levels of caring attributes [25]. One of the reasons for this discrepancy is that the author surveyed only undergraduate students and the participants had no service records and experience of independent care in the clinic. Also, Mahul et al. (2022) showed a positive relationship between age and the caring ability of students. One of the reasons for this discrepancy is that the tools used in the two studies were different [21].

### 4.1 Limitation

One of the limitations of the present study is that since the participants were selected from only one university and one educational system, and their number was relatively small, the generalizability of the findings is restricted. Another point is that the present study employed a descriptive‐comparative design and was conducted in a short period of time. However, caring attitudes and behaviors can be better explored through a longitudinal design which was not possible in the present study due to time constraints. Accordingly, it is recommended to use longitudinal studies to better capture changes in caring attributes of students.

## 5 Conclusion

Caring attributes reflect nurses' view of care and the importance of caring behaviors and roles for nurses. This study aimed to compare the caring attributes experienced by nursing students at undergraduate and postgraduate levels. In summary, while overall caring attributes were at a desirable level among the nursing students, significant differences emerged based on demographic factors and educational level. Specifically, female students exhibited higher caring attribute scores compared to their male counterparts. At the undergraduate level with eight semesters, nursing students in the fifth semesters have the highest caring attributes. PhD students demonstrated stronger caring communication skills. Also, the undergratuated students had the highest score in caring negation compared to another educational level. These findings suggest that nursing educators should tailor their teaching strategies to address gender‐based differences in caring attributes at the undergraduate level and leverage the advanced communication skills of PhD students as mentors. These findings also, help nursing educators optimize educational programs based on students' real needs and guide them toward cultivating stronger caring qualities. Further research is needed to explore the underlying factors contributing to these differences and to develop targeted interventions to enhance caring attributes across all student populations.

### 5.1 Implications for Nursing Education

Based on these findings, there are several practical applications for enhancing nursing education. First, educators and curriculum planners can design more targeted strategies to address gender differences in caring attributes. For example, specialized training programs for male students could help balance caring competencies across genders. Second, leveraging postgraduate students, such as PhD candidates, as mentors and role models can facilitate the transfer of advanced communication skills and caring behaviors, thus strengthening clinical interactions. Third, focusing on aspects like caring negation within educational programs can raise students' awareness of patients' needs and feelings. Overall, these insights can guide the development of tailored educational interventions, improve communication skills, and foster stronger caring attitudes among future nurses. Ultimately, aligning curricula more closely with students' real needs will help cultivate nurses who hold a deep appreciation for the value of caring, leading to an overall improvement in care quality within the healthcare system.

## Author Contributions

**Behnaz Bagherian:** conceptualization, investigation, writing – original draft, writing – review and editing, validation, methodology, formal analysis, software, supervision. **Monirsadat Nematollahi:** writing – review and editing, writing – original draft, supervision. **Roghayeh Mehdipour‐Rabori:** writing – original draft, writing – review and editing, supervision. **Shima Mehrabian:** data curation, writing – original draft, writing – review and editing. **Peiman Parandeh Afshar:** writing – original draft, writing — review and editing, data curation, formal analysis. **Asma Ghonchehpour:** formal analysis, writing – original draft, writing – review and editing, supervision, methodology, software.

## Consent

The authors have nothing to report.

## Conflicts of Interest

The authors declare no conflicts of interest.

## Transparency Statement

The lead author Asma Ghonchehpour affirms that this manuscript is an honest, accurate, and transparent account of the study being reported; that no important aspects of the study have been omitted; and that any discrepancies from the study as planned (and, if relevant, registered) have been explained.

## Data Availability

The data sets used and/or analyzed during the current study are available from the corresponding author on reasonable request. A. GH had full access to all of the data in this study and takes complete responsibility for the integrity of the data and the accuracy of the data analysis.
